# Supplementary material for: Fungal–Bacterial Networks in the Habitat of SongRong (Tricholoma matsutake) and Driving Factors of Their Distribution Rules
Source: J Fungi (Basel). 2022 May 27;8(6):575. doi: 10.3390/jof8060575 (PMC9225054; doi:10.3390/jof8060575)
Supplement: Supplementary file 1 [file jof-08-00575-s001.zip › jof-1729671-supplementary-Figure S1-S3.pdf]

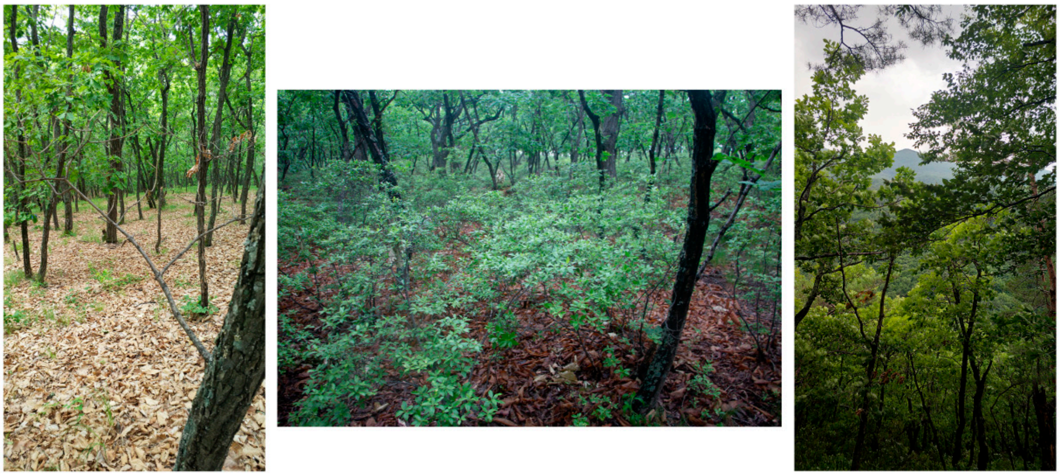

a b c

Figure S1: Habitat map of three sampling sites. (a) plot A covered by pure Quercus mongolica forest; (b) plot B is covered by Quercus mongolica mixed Rhododendron dauricum forest; (c) plot C is covered by Quercus mongolica mixed Rhododendron dauricum and Pinus densiflora forest.

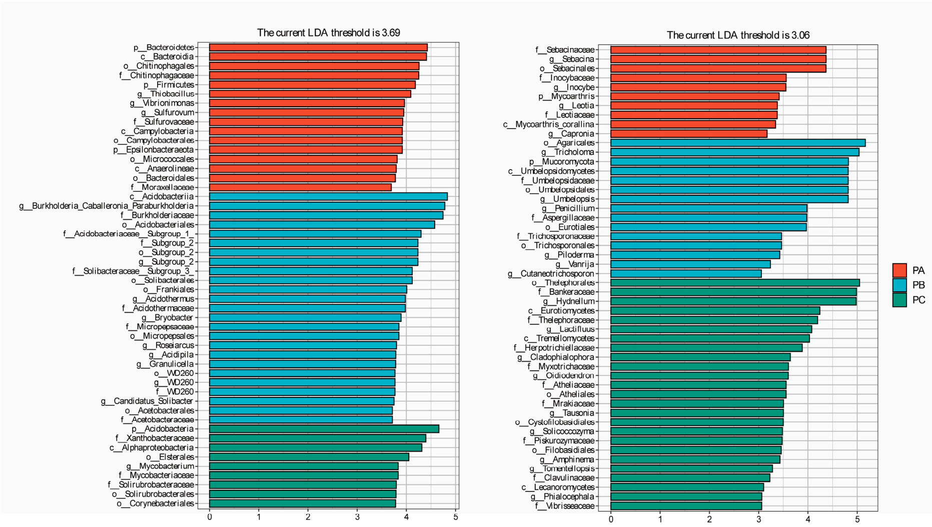

a b

Figure S2 Lefse analysis of fungi and bacteria in the soil from three plots. (a)Lefse analysis of bacteria in the soil from three plots. The default score of the LDA is 3.69, and the length of the bar chart represents the influence degree of the LDA score value and the species with significant differences between the different groups. (b) Lefse analysis of bacteria in the soil from three plots. The default score of the LDA is 3.06, and the length of the bar chart represents the influence degree of the LDA score value and the species with significant differences between the different groups. PA, soil samples from plot A; PB, soil samples from plot B; PC, soil samples from plot C.

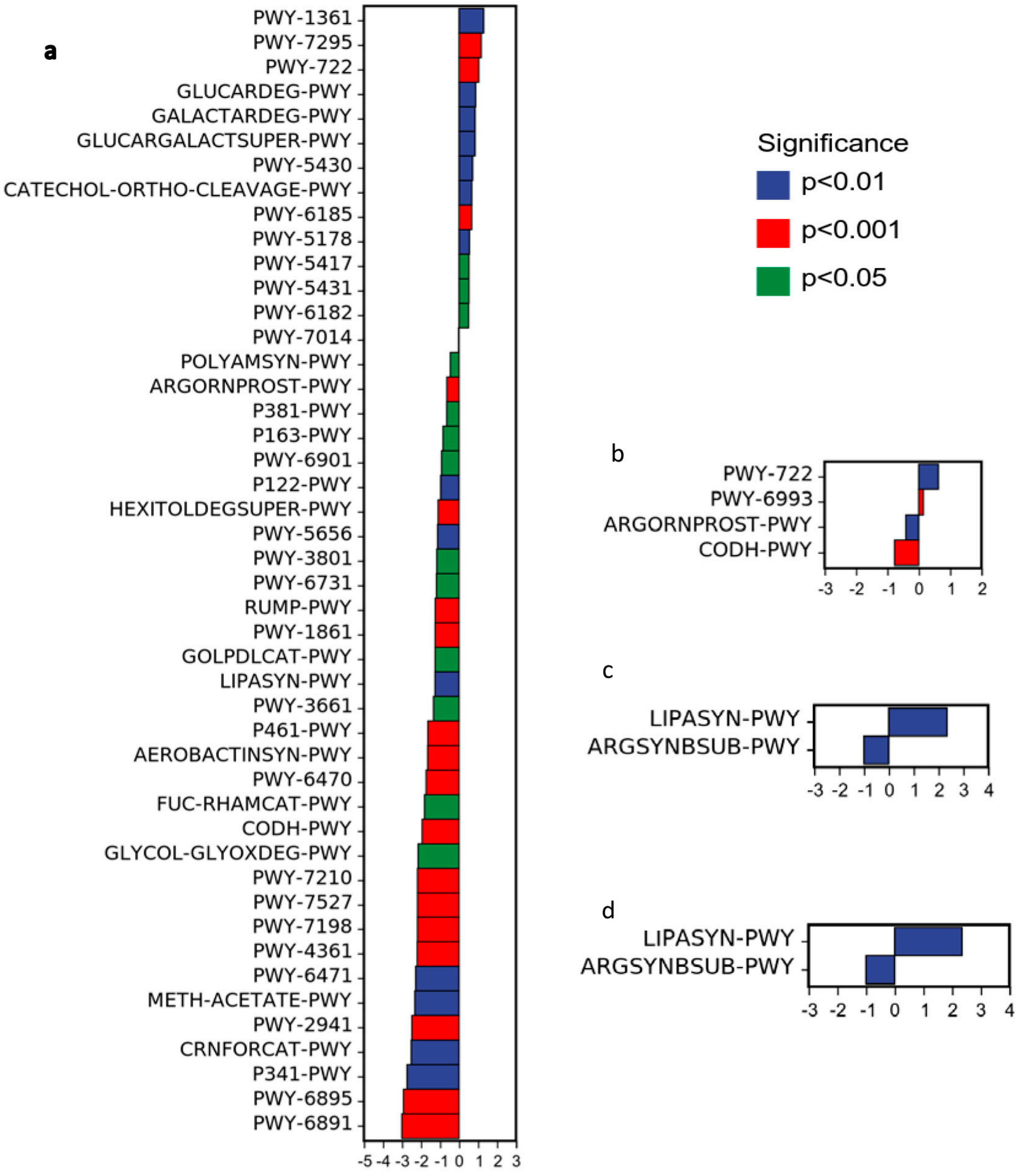

Figure S3 Differential metabolic pathways (a) Differential metabolic pathway of bacteria in the group of PB/PA; (b) Differential metabolic pathway of bacteria in the group of PC/PA; (c) Differential metabolic pathway of fungi in the group of PB/PA; (d)Differential metabolic pathway of fungi in the group of PC/PA.
