# Supplementary material for: Fungal–Bacterial Networks in the Habitat of SongRong (Tricholoma matsutake) and Driving Factors of Their Distribution Rules
Source: J Fungi (Basel). 2022 May 27;8(6):575. doi: 10.3390/jof8060575 (PMC9225054; doi:10.3390/jof8060575)
Supplement: Supplementary file 1 [file jof-08-00575-s001.zip › jof-1729671-supplementary-Table S1.pdf]

Table S1 The dominant bacterial genera and their relative abundance in the soil of three plots

Samples	Genus	The relative abundance
PA	*Subgroup_6*	4.34%
	Candidatus_Udaeobacter	3.57%
	Thiobacillus	2.86%
	KD4-96	2.27%
	Bradyrhizobium	2.11%
	Allorhizobium-Neorhizobium-Pararhizobium-Rhizobium	1.63%
	Subgroup_2	1.60%
	Mycobacterium	1.15%
	Sphingomonas	1.15%
	RB41	1.04%
PB	*Burkholderia-Caballeronia-Paraburkholderia*	12.62%
	Subgroup_2	5.25%
	Bradyrhizobium	3.26%
	Acidothermus	2.50%
	Bryobacter	2.48%
	Mycobacterium	1.91%
	Candidatus_Solibacter	1.78%
	Candidatus_Udaeobacter	1.75%
	Roseiarcus	1.70%
	AD3	1.60%
	Granulicella	1.48%
	Candidatus_Xiphinematobacter	1.34%
	Subgroup_6	1.30%
	Conexibacter	1.22%
PC	*Subgroup_2*	4.99%

Bradyrhizobium	3.96%
Subgroup_6	3.53%
Burkholderia-Caballeronia-Paraburkholderia	3.18%
Candidatus_Udaeobacter	2.63%
Mycobacterium	2.54%
Bryobacter	2.28%
Acidothermus	1.62%
Candidatus_Solibacter	1.45%
AD3	1.38%
KD4-96	1.38%
Sphingomonas	1.38%
RB41	1.18%
IMCC26256	1.10%
Conexibacter	1.03%
Roseiarcus	1.01%

Note: PA, the soil sample from the plot A (132°3′2.35″E, 46°7′20.19″N, 230m asl); PB, soil sample from the plot B (132°3′2.35″E, 46°7′20.19″N, 230m asl); PC, soil sample from the plot C (129°39′52.70″E, 42°33′31.75″N, 380 m asl).
