# Supplementary material for: Fungal–Bacterial Networks in the Habitat of SongRong (Tricholoma matsutake) and Driving Factors of Their Distribution Rules
Source: J Fungi (Basel). 2022 May 27;8(6):575. doi: 10.3390/jof8060575 (PMC9225054; doi:10.3390/jof8060575)
Supplement: Supplementary file 1 [file jof-08-00575-s001.zip › jof-1729671-supplementary-Table S2.pdf]

Table S2 The dominant fungal genera and species compositions of *Tricholoma*

(A) The dominant fungal genera and their relative abundance in the soil of three plots

Samples	Genus	The relative abundance
PA	*Russula*	48.72%
	Cortinarius	16.32%
	Mortierella	11.42%
	Sebacina	4.63%
	Tomentella	2.51%
PB	*Russula*	24.64%
	Tricholoma	21.84%
	Umbelopsis	13.48%
	Cortinarius	8.26%
	Hygrophorus	3.02%
	Sistotrema	2.56%
	Mortierella	2.46%
	Lactarius	2.37%
	Penicillium	2.02%
	Cenococcum	1.69%
	Trichoderma	1.34%
PC	*Russula*	32.34%
	Hydnellum	18.78%
	Mortierella	5.10%
	Umbelopsis	4.26%
	Delastria	3.36%
	Sebacina	2.53%
	Lactifluus	2.46%
	Tricholoma	1.87%
	Penicillium	1.77%
	Hygrophorus	1.49%
	Tomentella	1.40%
	Geminibasidium	1.34%
	Cladophialophora	1.12%
	Saitozyma	1.00%

(B) The relative abundance of species in the genus *Tricholoma*

**Species**	**the relative abundance**		
	**PA**	**PB**	**PC**
T. equestre	0.0000%	0.0000%	0.0000%
T. matsutake	0.0000%	0.4237%	0.0001%
T. portentosum	0.0000%	0.0009%	0.0097%
T. saponaceum	0.0000%	0.0068%	0.0002%

T. stans	0.0000%	0.0033%	0.0000%
T. ustaloides	0.0002%	0.0005%	0.0033%
Unclassified *Tricholoma*	0.0000%	0.0013%	0.0019%
unidentified	0.0000%	0.0000%	0.0213%

Note: PA, the soil sample from the plot A (132°3′2.35″E, 46°7′20.19″N, 230m asl); PB, soil sample from the plot B (132°3′2.35″E, 46°7′20.19″N, 230m asl); PC, soil sample from the plot C (129°39′52.70″E, 42°33′31.75″N, 380 m asl).
