# Supplementary material for: Fungal–Bacterial Networks in the Habitat of SongRong (Tricholoma matsutake) and Driving Factors of Their Distribution Rules
Source: J Fungi (Basel). 2022 May 27;8(6):575. doi: 10.3390/jof8060575 (PMC9225054; doi:10.3390/jof8060575)
Supplement: Supplementary file 1 [file jof-08-00575-s001.zip › jof-1729671-supplementary-Table S3.pdf]

Table S3 The spearman correlation analysis of the relative abundance of *Tricholoma* with other fungi in the habitat soil

Fungi genus in soil	Spearman correlation coefficient (r)	Significance(*p*)
Umbelopsis	0.946	0
Penicillium	0.919	0
Trichoderma	0.809	0
Solicoccozyma	0.78	0
Geomyces	0.749	0
Mucor	0.73	0.001
Apiotrichum	0.715	0.001
Leptodontidium	0.706	0.001
Gibberella	0.694	0.001
Saitozyma	0.692	0.001
Bifiguratus	0.676	0.002
Pseudogymnoascus	0.661	0.003
Cutaneotrichosporon	0.659	0.003
Absidia	0.658	0.003
Dasyspora	0.649	0.004
Trichocladium	0.631	0.005
Pseudotomentella	0.609	0.007
Cladophialophora	0.607	0.008
Vanrija	0.602	0.008
Russula	-0.832	0.001
Leotia	-0.781	0
Cortinarius	-0.717	0.013
Inocybe	-0.71	0.001
Trechispora	-0.695	0.001
Ilyonectria	-0.695	0.001

Entoloma	-0.666	0.003
Cylindrosympodium	-0.638	0.004
Clitocybe	-0.617	0.006
