# Supplementary material for: Fungal–Bacterial Networks in the Habitat of SongRong (Tricholoma matsutake) and Driving Factors of Their Distribution Rules
Source: J Fungi (Basel). 2022 May 27;8(6):575. doi: 10.3390/jof8060575 (PMC9225054; doi:10.3390/jof8060575)
Supplement: Supplementary file 1 [file jof-08-00575-s001.zip › jof-1729671-supplementary-Table S4.pdf]

Table S4 The spearman correlation analysis of the relative abundance of *Tricholoma* with bacteria genera in the habitat soil

Bacteria genus in soil	Spearman correlation coefficient (r)	Significance (*p*)
Terracidiphilus	0.875	0.000
Plot4-2H12	0.872	0.000
Burkholderia-Caballeronia-Paraburkholderia	0.866	0.000
Candidatus_Koribacter	0.854	0.000
Acidiphilium	0.849	0.000
Mucilaginibacter	0.843	0.000
Jatrophihabitans	0.826	0.000
Edaphobacter	0.816	0.000
GAS113	0.8	0.000
1174-901-12	0.796	0.000
Cupriavidus	0.794	0.000
Heliimonas	0.768	0.000
Inquilinus	0.746	0.000
Acidocella	0.733	0.001
Sporichthya	0.724	0.001
Coxiella	0.723	0.001
Gemmatirosa	0.718	0.001
Rhodanobacter	0.708	0.001
Actinoplanes	0.704	0.001
Crossiella	0.687	0.002
Kutzneria	0.681	0.002
Acidothermus	0.68	0.002
Acidicapsa	0.679	0.002

Legionella	0.679	0.002
Bradyrhizobium	0.676	0.002
Candidatus_Berkiella	0.672	0.002
Labrys	0.67	0.002
Ktedonobacter	0.667	0.002
Roseiarcus	0.662	0.003
bacterium_Ellin6515	0.652	0.003
Pedosphaera	0.65	0.004
bacterium_Ellin516	0.65	0.003
Nevskia	0.648	0.004
Candidatus_Solibacter	0.645	0.004
Acidipila	0.63	0.005
Aquicella	0.628	0.005
Granulicella	0.626	0.005
bacterium_enrichmentculturecloneauto67_4W	0.622	0.006
bacterium_enrichmentculturecloneheteroA49_4W	0.616	0.006
Occallatibacter	0.614	0.007
Mycobacterium	0.603	0.008
Actinospica	0.603	0.008
Phyllobacterium	-0.835	0
Pseudoxanthomonas	-0.791	0
Streptococcus	-0.761	0
Nitrospira	-0.749	0
Thermomonas	-0.717	0.001
Luteimonas	-0.655	0.003
Sphingobium	-0.638	0.004

MND1	-0.633	0.005
Bosea	-0.632	0.005
Taibaiella	-0.618	0.006
Agromyces	-0.611	0.007
