# Supplementary material for: Fungal–Bacterial Networks in the Habitat of SongRong (Tricholoma matsutake) and Driving Factors of Their Distribution Rules
Source: J Fungi (Basel). 2022 May 27;8(6):575. doi: 10.3390/jof8060575 (PMC9225054; doi:10.3390/jof8060575)
Supplement: Supplementary file 1 [file jof-08-00575-s001.zip › jof-1729671-supplementary-Table S5 Statistical tables of metab.pdf]

Table S5 Statistical tables of metabolic pathways based on MetaCyc database

(a) The metabolic pathways of soil bacteria in three sites

Level1	Level2	Abundance
Biosynthesis 12	Amine and Polyamine Biosynthesis	1131.4
	Amino Acid Biosynthesis	35287.95
	Aminoacyl-tRNA Charging	985.01
	Aromatic Compound Biosynthesis	2338.04
	Carbohydrate Biosynthesis	12309.05
	Cell Structure Biosynthesis	6908.41
	Cofactor, Prosthetic Group, Electron Carrier, and Vitamin Biosynthesis	33059.85
	Fatty Acid and Lipid Biosynthesis	22532.79
	Metabolic Regulator Biosynthesis	534.24
	Nucleoside and Nucleotide Biosynthesis	28217.32
	Other Biosynthesis	755.91
	Secondary Metabolite Biosynthesis	4899.2
Degradation/Utilization/Assimilation 16	Alcohol Degradation	301.95
	Aldehyde Degradation	53.34
	Amine and Polyamine Degradation	2165.35
	Amino Acid Degradation	3617.67
	Aromatic Compound Degradation	6899.45
	C1 Compound Utilization and Assimilation	3142.99
	Carbohydrate Degradation	4899.44
	Carboxylate Degradation	4390
	Chlorinated Compound Degradation	211.47
	Cofactor, Prosthetic Group, Electron Carrier Degradation	1.05
	Degradation/Utilization/Assimilation - Other	407.33
	Fatty Acid and Lipid Degradation	1693.49
	Inorganic Nutrient Metabolism	3960.96
	Nucleoside and Nucleotide Degradation	5965.32
	Polymeric Compound Degradation	1942.72
	Secondary Metabolite Degradation	4169.72
Detoxification2	Antibiotic Resistance	110.13
	methanol oxidation to carbon dioxide	193.68
Generation of Precursor Metabolite and Energy17	1,5-anhydrofructose degradation	45.11
	Electron Transfer	3478.55
	Entner-Duodoroff Pathways	18.69

	ethylmalonyl-CoA pathway	164.95
	Fermentation	9323.24
	formaldehyde oxidation I	37.3
	Glycolysis	3060.28
	glyoxylate cycle	913.46
	isopropanol biosynthesis	256.18
	methyl ketone biosynthesis	195.58
	methylaspartate cycle	100.59
	Pentose Phosphate Pathways	2738.17
	Photosynthesis	1890.84
	Respiration	3563.85
	superpathway of glycolysis and Entner-Doudoroff	931.8
	superpathway of glycolysis, pyruvate dehydrogenase, TCA, and glyoxylate bypass	975.15
	TCA cycle	8156.17
Glycan Pathways2	Glycan Biosynthesis	776.92
	Glycan Degradation	1080.88
Macromolecule Modification2	Nucleic Acid Processing	1476.53
	Protein Modification	0.01
Metabolic Clusters10	L-glutamate and L-glutamine biosynthesis	481.04
	O-antigen building blocks biosynthesis (E. coli)	1065.07
	phospholipases	16.89
	pyrimidine deoxyribonucleotide phosphorylation	991.8
	pyrimidine deoxyribonucleotides biosynthesis from CTP	6.39
	pyrimidine deoxyribonucleotides de novo biosynthesis I	1008.79
	pyrimidine deoxyribonucleotides de novo biosynthesis III	518.52
	pyrimidine deoxyribonucleotides de novo biosynthesis IV	4.48
	superpathway of L-aspartate and L-asparagine biosynthesis	423.49
	tRNA charging	985.01

(b) The metabolic pathways of soil fungi in three sites

Level1	Level2	Abundance
Biosynthesis7	Amino Acid Biosynthesis	3105.23
	Aminoacyl-tRNA Charging	914.9
	Carbohydrate Biosynthesis	2374.26
	Cofactor, Prosthetic Group, Electron Carrier, and Vitamin Biosynthesis	4611.78
	Fatty Acid and Lipid Biosynthesis	2433.44
	Nucleoside and Nucleotide Biosynthesis	8015.38
	Secondary Metabolite Biosynthesis	1629.24
Degradation/Utilization/Assimilation7	Amino Acid Degradation	461.18
	C1 Compound Utilization and Assimilation	684.31
	Carbohydrate Degradation	1126.14
	Degradation/Utilization/Assimilation - Other	736.47
	Fatty Acid and Lipid Degradation	2529.4
	Inorganic Nutrient Metabolism	1218.49
	Nucleoside and Nucleotide Degradation	859.27
Generation of Precursor Metabolite and Energy9	chitin degradation to ethanol	400.58
	Electron Transfer	5845.37
	Fermentation	1399.32
	Glycolysis	680.77
	glyoxylate cycle	1314.64
	methyl ketone biosynthesis	808.45
	Pentose Phosphate Pathways	1758.79
	Respiration	5845.37
	TCA cycle	1051.59
Glycan Pathways1	Glycan Biosynthesis	785.24
Metabolic Clusters5	phospholipases	51.14
	pyrimidine deoxyribonucleotide phosphorylation	693.88
	pyrimidine deoxyribonucleotides biosynthesis from CTP	423.63
	pyrimidine deoxyribonucleotides de novo biosynthesis I	852.93
	tRNA charging	914.9
